# Assessing risks of invasion through gamete performance: farm Atlantic salmon sperm and eggs show equivalence in function, fertility, compatibility and competitiveness to wild Atlantic salmon

**DOI:** 10.1111/eva.12148

**Published:** 2014-03-11

**Authors:** Sarah E Yeates, Sigurd Einum, Ian A Fleming, William V Holt, Matthew JG Gage

**Affiliations:** 1 School of Biological Sciences, University of East Anglia, Norwich Research ParkNorwich, UK; 2 Centre for Biodiversity Dynamics, Department of Biology, Norwegian University of Science and TechnologyTrondheim, Norway; 3 Norwegian Institute for Nature ResearchTrondheim, Norway; 4 Department of Ocean Sciences, Memorial University of NewfoundlandNewfoundland, Canada; 5 Academic Department of Reproductive and Developmental Medicine, University of SheffieldSheffield, UK

**Keywords:** aquaculture, fertilization, gamete, salmon, sperm competition

## Abstract

Adaptations at the gamete level (a) evolve quickly, (b) appear sensitive to inbreeding and outbreeding and (c) have important influences on potential to reproduce. We apply this understanding to problems posed by escaped farm salmon and measure their potential to reproduce in the wild. Farm Atlantic salmon (*Salmo salar*) are a threat to biodiversity, because they escape in large numbers and can introgress, dilute or disrupt locally adapted wild gene pools. Experiments at the whole fish level have found farm reproductive potential to be significant, but inferior compared to wild adults, especially for males. Here, we assess reproductive performance at the gamete level through detailed *in vitro* comparisons of the form, function, fertility, compatibility and competitiveness of farm *versus* wild Atlantic salmon sperm and eggs, in conditions mimicking the natural gametic microenvironment, using fish raised under similar environmental conditions. Despite selective domestication and reduced genetic diversity, we find functional equivalence in all farm fish gamete traits compared with their wild ancestral strain. Our results identify a clear threat of farm salmon reproduction with wild fish and therefore encourage further consideration of using triploid farm strains with optimized traits for aquaculture and fish welfare, as triploid fish remain reproductively sterile following escape.

## Introduction

Biologists now recognize that processes at the level of the gamete can have profound effects upon reproductive success and gene flow, especially within promiscuous mating systems where sperm competition and sperm–egg compatibility systems can influence fertilization success (reviewed in Birkhead et al. 2009). We also recognize that traits involved in fertilization can evolve extremely rapidly, with proteins controlling sperm–egg associations being some of the fastest evolving traits so far measured (Swanson and Vacquier 2002). We therefore apply this evolutionary knowledge about (i) the importance of gamete performance and (ii) the speed of gamete trait evolution, to improve our understanding of the risks of reproduction between wild Atlantic salmon (*Salmo salar*) and escaped domesticated fish from farms. Salmon farming has increased exponentially since the late 1960s (Tilseth et al. 1991), and it is estimated that more than 95% of adult Atlantic salmon in existence today on our planet are domestically selected farm fish (Naylor et al. 2005). Global production is currently estimated at around 2 million tonnes per annum, a 30% increase on the previous 5-year average (ICES WGNAS 2013), and the worldwide production of farmed Atlantic salmon is more than 1300 times the reported catch of wild fish from the North Atlantic (ICES WGNAS 2013). Because of this scale of production, huge numbers of farmed salmon escape (during routine handling and large-scale accidents) from both freshwater and marine aquaculture at all life stages (reviewed in Gross 1998; Naylor et al. 2005; Thorstad et al. 2008). McGinnity et al. (2003) estimated that around 2 million farmed fish escaped into the North Atlantic annually, comprising ∼50% of the total prefishery abundance of wild salmon in the area, and Naylor et al. (2005) reported that 20–40% of salmon caught in the North Atlantic seas off the Faroes between 1989 and 1996 were of farmed origin. Although the rate of farm escapees has decreased (Ferguson et al. 2007; Thorstad et al. 2008; Jensen et al. 2010), the enormity of salmon farming continues to grow, so risks to wild Atlantic salmon from aquaculture require attention. The wild Atlantic salmon is a fish species with major biological, ecological and commercial significance (Verspoor et al. 2007; Aas et al. 2011), but natural populations are in major decline, with the total nominal catch across the North Atlantic currently at its lowest levels, and reduced by almost 90% since the 1970s (ICES WGNAS 2013). One serious threat to wild salmon populations comes from aquaculture escapes, which are known to occur in sufficiently large numbers to present a risk of farm fish reproduction and therefore introgression (Hutchings 1991; Gross 1998; Fleming et al. 2000; McGinnity et al. 2003; Naylor et al. 2005; Hindar et al. 2006; Ferguson et al. 2007; Thorstad et al. 2008; Glover et al. 2012). Once on wild spawning grounds, farm salmon can reproduce with wild fish (Lura and Saegro 1991; Webb et al. 1991), with population-level evidence of genetic introgression (Skaala et al. 2006, Glover et al. 2012). Farm salmon have major genetic differences to wild populations because (i) farm strains are almost always derived from nonlocal sources, so lack locally adapted alleles which are especially important in salmon (Gjedrem et al. 1991; Thorstad et al. 2008), and (ii) because decades of selective domestic breeding have resulted in significantly altered and reduced allelic diversity, making them generally not adapted to the wild (Fleming et al. 1997, Youngson et al. 2001, McGinnity et al. 2003; Thorstad et al. 2008).

Farm fish reproduction, if continual, threatens the long-term integrity of wild Atlantic salmon populations through genetic swamping causing dilution and erosion of local adaptations (Hindar et al. 2006; Ferguson et al. 2007; Thorstad et al. 2008). A recent study focusing on the effect of farm genotypes on Atlantic salmon populations near the southern end of their range exemplifies how hybridization with farm strains can disrupt the phenology of locally adapted populations (Fraser et al. 2010). On top of genetic disruption, is the ecological load arising from juveniles carrying farm genes: selection for rapid growth and efficient feed conversion in crowded conditions has created a farmed phenotype that can show elevated aggression, decreased response to predation and altered phenology compared with wild phenotypes (Einum and Fleming 1997; Fleming and Einum 1997; McGinnity et al. 2003; Fraser et al. 2010). These characteristics create the paradoxical situation whereby offspring of farm fish may aggressively out-compete wild juveniles for territories and food, but in so doing expose themselves to elevated risks that reduce their longer-term fitness (Fleming et al. 2000; Einum and Fleming 2001; McGinnity et al. 2003; Thorstad et al. 2008). In an experimental study where farm and wild Atlantic salmon were simultaneously released into one controlled river system, farm fish depressed the productivity of seaward migrants from wild counterparts by over 30% (Fleming et al. 2000).

There is therefore a need to understand the risks, at all levels, of reproduction between farm and wild Atlantic salmon in order to assess the probability of introgression and genetic swamping. Experimental work shows that farm fish reproductive performance is not equivalent to that of wild salmon, with reduced reproductive performance particularly by males (Crozier 1993; Fleming et al. 1996, 2000; Carr et al. 1997; Weir et al. 2004). For example, in the experimental release of farm and wild salmon mentioned above, the farm fish achieved 28% the breeding success (=embryos reproduced) of wild fish, and over a full generation, adult to adult, 16% that of wild fish (Fleming et al. 2000). The main ‘bottleneck’ to farm fish invasion to the wild was thus breeding success. Some of the inferior reproductive performance of farm salmon arises from behavioural inadequacies, such as reduced or inappropriate spawning behaviours, fewer nests dug or covered and more retained gametes. As Atlantic salmon spawn polyandrously (Fleming 1996; Jordan et al. 2007; Weir et al. 2010), it is relevant to measure reproductive performance in the context of intrasexual competition, where farm males show poor performance (Fleming et al. 1996; Weir et al. 2004).

By contrast with work on the behavioural ecology of farm Atlantic salmon reproduction, far less is known about the form, function and fertility of farm salmon sperm and eggs compared with wild fish. This is an important gap in our knowledge, because processes at the gamete level can have consequential effects upon reproductive success (Birkhead et al. 2009), especially within a polyandrous mating system (Weir et al. 2010). Moreover, if farm escapees become ‘naturalized’ after a period following escape into the wild and therefore develop more appropriate reproductive behaviours when they find a spawning system, it will be important to determine whether their gametes are fully functional. We hypothesize that three important processes from aquaculture could have changed farm salmon gamete performance when crossing with wild fish. First, because hatchery breeding usually involves artificial fertilization using stripped or even cryopreserved gametes under conditions that are very different to the wild, this could have relaxed selection on gamete traits required for success within natural spawnings, a phenomenon recognized in other domesticated strains where selection has focused only on specific traits (e.g. cattle, Weigel 2006). Second, because Atlantic salmon domestication has led to intentional and unintentional selection for farm-friendly phenotypes (Gross 1998; Fleming and Einum 1997, Youngson et al. 2001, McGinnity et al. 2003; Thorstad et al. 2008), sperm–egg recognition and compatibility systems that evolve to encourage local adaptation (Yeates et al. 2009) or avoid hybridization (Yeates et al. 2013) could be disrupted by aquaculture. Third, because line breeding for domestication can also result in the loss of genetic diversity, which we know can lead to negative impacts upon gamete traits through inbreeding depression (Fitzpatrick and Evans 2009).

Wild Atlantic salmon are naturally polyandrous (Jordan et al. 2007), with females being fertilized by up to 16 males in one spawning bout (Weir et al. 2010); thus, sperm competition will be a prevalent phenomenon for individual reproductive success. In Atlantic salmon, we know that sperm traits are important for fertilization (Yeates 2005) and essential for sperm competition success (Gage et al. 2004; Vladić et al. 2010). We also know that significant natural variation in sperm performance exists (Gage et al. 2004; Vladić et al. 2010), even to the extent of enabling ‘sneaky’ male mating tactics, otherwise disfavoured by females as spawning partners, to persist as evolutionary stable strategies through a significantly improved performance in sperm competition (Gage et al. 1995; Vladić et al. 2010). From the female perspective, we know that the ovum does not play a passive role in fertilization and that chemoattraction to the micropyle is important for fertilization (Yanagimachi et al. 1992, Yanagimachi et al. 2013) and potentially cryptic female choice (Turner and Montgomerie 2002; Rosengrave et al. 2008; Yeates et al. 2009, Butts et al. 2012, Yeates et al. 2013). These processes operating at the level of the gamete can therefore have significant influence on reproductive outcomes and need to be considered if we are to understand the full risk of farm Atlantic salmon reproduction in the wild.

Few studies have examined how aquaculture impacts on fertility and rarely with regard to the natural mating pattern which can generate sperm competition and/or mechanisms influencing sperm–egg compatibility. In farmed *Penaeus* prawns, pond-reared males have poor sperm quality and problematically lowered fertility, compared with wild relatives (Leung-Trujillo and Lawrence 1987; Alfaro and Lozano 1993; Pratoomchat et al. 1993). In first-generation farmed cod (*Gadus morhua*), males showed reduced sperm quality compared with wild equivalents, especially at the start of the breeding season, and this translated into inferior sperm fertility and competitiveness, possibly mediated by diet (Skjæraasen et al. 2009; Butts et al. 2011). In haddock (*Melanogrammus aeglofinus*), however, cultured and wild males showed equivalent sperm motility and concentration (Rideout et al. 2004), and in sea trout (*Salmo trutta*), sperm densities between wild and sea-reared males showed differences that were opposite between years (Poole and Dillane 1998). By contrast with the studies in these aquaculture species, however, farm Atlantic salmon have been subjected to almost 50 years of selective domestication since the 1970s (Tilseth et al. 1991) so that genetic influences from relaxed selection, directed domestication and reduced genetic diversity could be influential phenomena. Only one study has examined sperm trait differences in an aquaculture species that has experienced multiple generations of domestic selection: comparisons between farm and wild Chinook salmon revealed that farm males produce sperm with significantly higher sperm concentration, motility, longevity and velocity compared with wild males (Lehnert et al. 2012), revealing clear potential for fertilization success and therefore introgression after escape. Compared with Chinook, Atlantic salmon aquaculture is a far bigger commercial activity (with more escapees) across a huge geographical range and associated with a longer history of more intense domestic breeding and clear evidence of genetic change (e.g. Glover et al. 2011). We therefore conducted a series of detailed assays on sperm and egg form and function, allowing us to compare the performance of gametes from farm salmon with their wild ancestors. To identify genetic differences in gamete traits between farm and wild fish, and therefore make predictions about reproduction following loss to the wild, we compare traits from fish raised under similar environmental conditions, therefore equalizing any effects on reproductive performance of, for example, diet (Skjæraasen et al. 2009). In addition to detailed measures of sperm number and motility, we conduct a full assessment of gamete performance in (i) fertilization, (ii) sperm competition and (iii) measures of sperm–egg compatibility between farm and wild gametes. Because salmon spawn externally, our measures of gamete function can be conducted in the natural micro-environment to which sperm and eggs are adapted, thereby allowing a relevant assessment of the risks of farm Atlantic salmon reproduction and subsequent introgression to wild gene pools.

## Methods

### Field site and fish groups

Fish maintenance, fertilization trials and egg rearing were carried out at the Norwegian Institute of Nature Research (NINA) Aquatic Research Station in Ims, Norway. Fish were maintained and handled according to standard hatchery protocols approved by the Norwegian Animal Research Authority. The wild adult Atlantic salmon were from the river Namsen (Norway), and the farmed fish were seventh generation from Norway's national breeding programme Aquagen, Sunndalsøra. The Aquagen strain used in the present study originates predominantly from fish of the River Namsen (Gjedrem et al. 1991; Garant et al. 2003). Gametes were recovered from adult fish that had been hatched and reared in the hatchery at Ims, so fish experienced similar environmental backgrounds, and the hatchery rearing allowed close monitoring of multiple adults entering breeding condition so that we were able to source ripe males and females of both farm and wild strains for simultaneous *in vitro* fertilization and competition experiments. Adult fish were maintained in single-strain groups in 4000 L tanks fed directly by natural river Imsa water, and the fish used in the experiments were all 3 years of age and size matched. At the onset of the spawning season, fish were checked daily, and gametes stripped from those showing full reproductive condition with free-running eggs or semen, using standard hatchery procedures (Gage et al. 2004; Yeates 2005; Yeates et al. 2009). Stripped gametes were stored before experimentation for a maximum of 3 days on wet ice just above 0°C in airtight, oxygenated bags. All activations and recordings of sperm motility, and fertilization and sperm competition trials, were performed at the natural river water temperature of 3°C and in an air temperature of 3–4°C and within 3 days of strip. Checks on gamete performance after storage showed no change under these conditions (Yeates et al. 2013), and as all fish were stripped on the same day, then examined in experimental groups comparing both farm and wild sperm and eggs, there was no possibility of directional confounds on either farm or wild fish identity from time-since-strip. Prior to analysis or use in fertilization or competition trials, semen subsamples were diluted in Trout Extender (80 mm NaCl, 40 mm KCl, 1 mm CaCl_2_ and 20 mm Tris, adjusted to *pH* 9 (Billard and Cosson 1992) at a 1:1 ratio. This procedure predilutes the semi-viscous semen so that sperm are simultaneously and evenly activated on contact with water (Billard and Cosson 1992).

### Sperm trait analyses: concentration and morphometry

Sperm counts were conducted according to established protocols (Gage et al. 1998) using improved Neubauer chamber haemocytometers and multiplying the average of 4 separate counts by the sample's dilution factor. Sperm densities from *n* = 18 wild and 18 farm males were calculated within 20 h of strip. Sperm morphometric measures also followed established methods (Gage et al. 1998), with 5 *μ*L of sperm-extender subsamples being preserved before activation in 400 *μ*L of 5% formalin. Five microlitre of the preserved sample was then smeared onto a glass slide and air-dried, encouraging sperm to lie flat in a two-dimensional plane. Once dry, the slides were gently rinsed twice in distilled water to remove any crystalline residue. The resulting dried smear produces clear 2-dimensional images of the sperm cells under ×600 dark-field phase contrast, allowing capture and measurement with Olympus analySIS (Soft Imaging System gMBh, Münster, Germany). Flagellum length, head length and total sperm length were measured for 10 sperm per male using Scion Image (Scion Corporation, Frederick, MD, USA). Cumulative error testing, and the significant variance that occurs between (but not within) males in Atlantic salmon (and other taxa) mean that *n* = 10 sperm can accurately represent each male's mean sperm length (Gage et al. 1998; Morrow and Gage 2001). Sperm lengths from *n* = 14 wild and 14 farm males were measured.

### Sperm trait analyses: motility, velocity, linearity and longevity

To measure differences in sperm activity between farm and wild Atlantic salmon, we employed computer-assisted sperm analysis (CASA) optimized for fish (Kime et al. 2001) to compare behaviour of sperm activated in river water for *n* = 18 farm and *n* = 18 wild Atlantic salmon. Sperm-extender solutions were activated in river water at 3°C, then 0.7 *μ*L of the activated diluent rapidly transferred onto a 12-well multitest glass slide (ICN Basingstoke, UK) (well depth 0.0116 mm) and a round cover slip immediately put in place (Yeates 2005). Multiwell slides and a diluent volume of 0.7 *μ*L were optimum for recording a stable image that was free of general drift. The volume ratio of sperm-extender to river water was adjusted between 3 and 6 *μ*L to 400 mL water depending on concentration so that 50–100 spermatozoa were visible in the field of view at 400 ×  magnification for each trial (Gage et al. 2004; Yeates 2005). Time from activation, transfer onto slide, cover slip placement and initial recording was minimized to 3 s in order to capture as much of the sperm activation process as possible. A single recording of sperm activity was conducted for each male, any activation-to-recording procedure that took longer than 3 s, or if the image showed drift or was not focused (because of slight variation in slide thickness), then the procedure was repeated.

Sperm activity was recorded onto Sony Hi8 video tapes from a JVC video camera (TK-1280E) fixed to an Olympus CK40 inverted stage microscope at 400 × under dark-field phase illumination. Using CASA, we measured: (i) % motility (=the proportion of visible sperm showing forward motile progression), (ii) curvilinear velocity (=average sperm swimming speed: the average speed of progression along sperm swimming paths), (iii) longevity (=the active lifespan of the sperm sample, measured manually as the time at which all sperm visible in the microscope field of view ceased forward swimming progression) and (iv) linearity or straightness [=sperm swimming trajectories, measured as the average proportion derived from the ratio between the total trajectory distance swum *versus* the straight-line distance between the start and end of the path, and where perfect straightness = 1.0 (Kime et al. 2001; Yeates 2005)].

Sperm motility was measured through analysis of the Hi8 video tapes by CASA using a Hobson Sperm Tracker (Hobson Vision Ltd, Baslow, UK). Salmonid sperm typically show rapid swimming velocity over a brief lifespan (under 30–60 s (Yeates 2005; Yeates et al. 2007), so tracking data on % motility, curvilinear velocity and path straightness were collected for 15 s from 5 s after the time of sample activation (Kime et al. 2001). Longevity was the period from activation until sperm ceased forward progressive motility. The Hobson tracker was set to operate at a frame rate of 50 Hz, and the ‘minimum track point’ setting was 50 frames. The ‘search radius’ used was 8.13–10.56 *μ*m, and the ‘threshold’ set to +30/−100 with the objective at 40 × . None of the sperm trait data sets departed from a normal distribution (Kolmogorov–Smirnov tests all *P* > 0.216), so sperm trait data were compared between farm and wild fish using unpaired *t-*tests.

### Fertilization trials

Sperm and eggs were available for trials from 36 males and 36 females (18 + 18 farm and 18 + 18 wild) in sufficient amounts to split clutches and ejaculates and replicate across four cross-combinations: (i) farm ♂ X wild ♀ (*n* = 18), (ii) farm ♂ X farm ♀ (*n* = 18), (iii) wild ♂ X wild ♀ (*n* = 18), (iv) wild ♂ X farm ♀ (*n* = 18). Results from these different crosses enabled us to compare relative fertility from both the egg and the sperm perspective and also to employ a paired design (split clutches within individual females) for analysing between-*versus* within-strain fertilization compatibility.

All *in vitro* fertilizations took place in dry 1-L plastic beakers, with batches containing an average of 66 eggs (± 0.596SE, *n* = 360) exposed to a 5-*μ*L sperm-extender sample activated by 500 mL of Imsa river water (at natural temperatures of 3°C). This volume ratio of sperm/water was chosen to avoid ceiling effects from sperm saturation and instead create conditions where intermediate fertilization successes were achieved, allowing variation in relative fertility to be measured (Yeates 2005).

In addition to measuring relative fertility between farm and wild salmon through sperm number limitation, we also created experimental conditions where activated gametes were exposed to each other within limited time windows of 1, 2, 5, 20 and 180 s. These time limits were selected because (i) the association between eggs and sperm occurs rapidly in salmon, with just a two-second difference significantly affecting sperm competition success (Hoysak and Liley 2001; Gage et al. 2004; Yeates et al. 2007, Yeates et al. 2013), and (ii) Atlantic salmon sperm start to lose motility after 20–30 s from activation (Yeates 2005). Egg batches were held in 8 cm^3^ plastic boxes perforated all over with 3–4 mm holes, which enabled full mixing of the eggs with activated sperm, but also allowed control of time limitation by removing the eggs from the activated sperm mix followed by three river water-only rinses. The procedure followed thus: (i) activate 50 *μ*L sperm-extender using 500 mL of Imsa river water in 1 L beaker, (ii) within 2 s of activation (by which time all sperm are active Billard and Cosson 1992; Yeates 2005), immerse perforated container holding egg batch for either 1, 2, 5 or 20 s into the activated sperm medium, (iii) remove egg batch container and immediately immerse in three 1-L beakers containing river water only to rinse off any active sperm. In addition to the four time limits, we also ran a fifth, non-time-limited 180-s treatment (which is beyond the maximum sperm survival time in Atlantic salmon, Yeates 2005).

Each of the four cross-combinations, (i) farm ♂ X wild ♀ (*n* = 18), (ii) farm ♂ X farm ♀ (*n* = 18), (iii) wild ♂ X wild ♀ (*n* = 18) and (iv) wild ♂ X farm ♀ (*n* = 18), was replicated across the five different gamete exposure times (total *n* = 360 trials). After fertilization trials, egg batches were allowed to develop in uniquely coded trays in flow-through incubation channels with constant river water at natural temperatures (Gage et al. 2004; Yeates et al. 2013). Fertilization success was scored 15 d after the trials (water temperature was 3–4°C), by soaking eggs in 5% acetic acid, allowing visualization of developing embryos and scoring under 10 × magnification (Yeates 2005; Yeates et al. 2013).

The *n* = 72 non-time-limited 180 s fertilization trials were used to measure (a) whether egg fertility differed between farm and wild females (under sperm limitation, and with either farm or wild males, or both), and whether any fertilization incompatibility had developed through selective domestic breeding. Fertilization successes in the 180-s time blocks showed normal distributions (Kolmogorov–Smirnov tests all *P* > 0.131), allowing farm and wild egg fertility to be compared using unpaired *t-*tests, and egg–sperm compatibility to be compared (between split clutches within individual females) using paired *t-*tests. Time-limited fertilization success data were analysed using repeated-measures anova, exploring the simultaneous effects of strain and gamete exposure time. As all of the data for the 1-s exposure time showed departures from normality due to high frequencies of zero success (which could not be transformed), we also ran a second repeated-measures (RM) anova without this time block. In addition, we also ran separate nonparametric unpaired Mann–Whitney comparisons between farm and wild male fertility within each time block. All three analyses presented the same picture.

### Sperm competition trials

In addition to measures of relative fertility, we examined whether sperm competitiveness differed between farm and wild Atlantic salmon. Similar *in vitro* protocols were followed to the fertilizations where gametes were mixed with 500 mL of river water in 1 L beakers, except that egg batches were instead fertilized by a mix of 40 *μ*L sperm-extender from a farm male and 40 *μ*L sperm-extender from a wild male, homogenized by gently and repeatedly drawing the sperm-extender solutions in and out of an autopipette. DNA was preserved from fin clips in ethanol of all adults used. A total of 25 two-male competitions were run for eggs from *n *=* *14 farmed females, and *n *=* *11 wild females. Eggs were reared as previously described, allowing 2 months of development at which point embryos were preserved in ethanol for genetic analysis. The very low numbers of eggs which failed in normal development (<1%) meant that differential embryo mortality did not confound the overall findings. Sperm competition success of farm males was analysed using nonparametric Wilcoxon tests, comparing the observed number of successful fertilizations gained by farm males against the null expectation that farm and wild males shared an equal 50% of the paternity in each competition.

### Parentage analysis

Microsatellite DNA analysis was used to assign paternity of an average 25 offspring across the 25 competitions (range 14–31). DNA was extracted from the adult tissue and from a small piece of tissue of the developing embryo removed from inside the egg, using a modified salt extraction technique (Aljanabi and Martinez 1997) in 96-well plates (ABgene, Surrey, UK). Paternity was assigned to offspring using up to 2 noninterrupted microsatellite loci (*Ssa408* and *ssa410*) and one compound locus (*Ssa417*) (Cairney et al. 2000), which amplify and exhibit substantial polymorphism in Atlantic salmon (Aljanabi and Martinez 1997; Yeates et al. 2013). Once parental genotypes were known, often only a single locus was needed to unambiguously assign paternity in a 2-male competition. PCR amplification was carried out in a 10 *μ*L volume containing 1 *μ*L of template DNA (unspecified concentration); 5 *μ*L 2 × PCR Mastermix with 1.5 mm MgCl_2_ (Abgene), 1 *μ*L BSA (10 mg mL^−1^), 0.5 *μ*m labelled forward primer, 0.5 *μ*m reverse primer and sterile distilled water to total volume. Forward PCR primers were fluorescently labelled with FAM (Ssa408) HEX (Ssa410) and NED (Ssa421) (Applied Biosystems, South San Francisco, CA, USA). An initial 3-min denaturation at 94°C preceded 29 denaturing (94°C for 15 s), annealing (61°C for 15 s) and extension (72°C for 15 s) cycles. Annealing temperatures were 58°C for Ssa408 and Ssa421; and 53°C for Ssa410. Parentage was determined by comparing alleles at the locus or loci used, with alleles from the mother and both of the potential fathers. PCR products were run on an ABI3700 automated DNA sequencer with the Genescan-500 ROX-labelled size standard (Applied Biosystems). Fragment lengths were determined using the Genescan and Genotyper software packages v 3.7 (Applied Biosystems).

## Results

All *P* values ≤ 0.05 were considered to be biologically significant.

### Sperm traits

**Figure 1:**
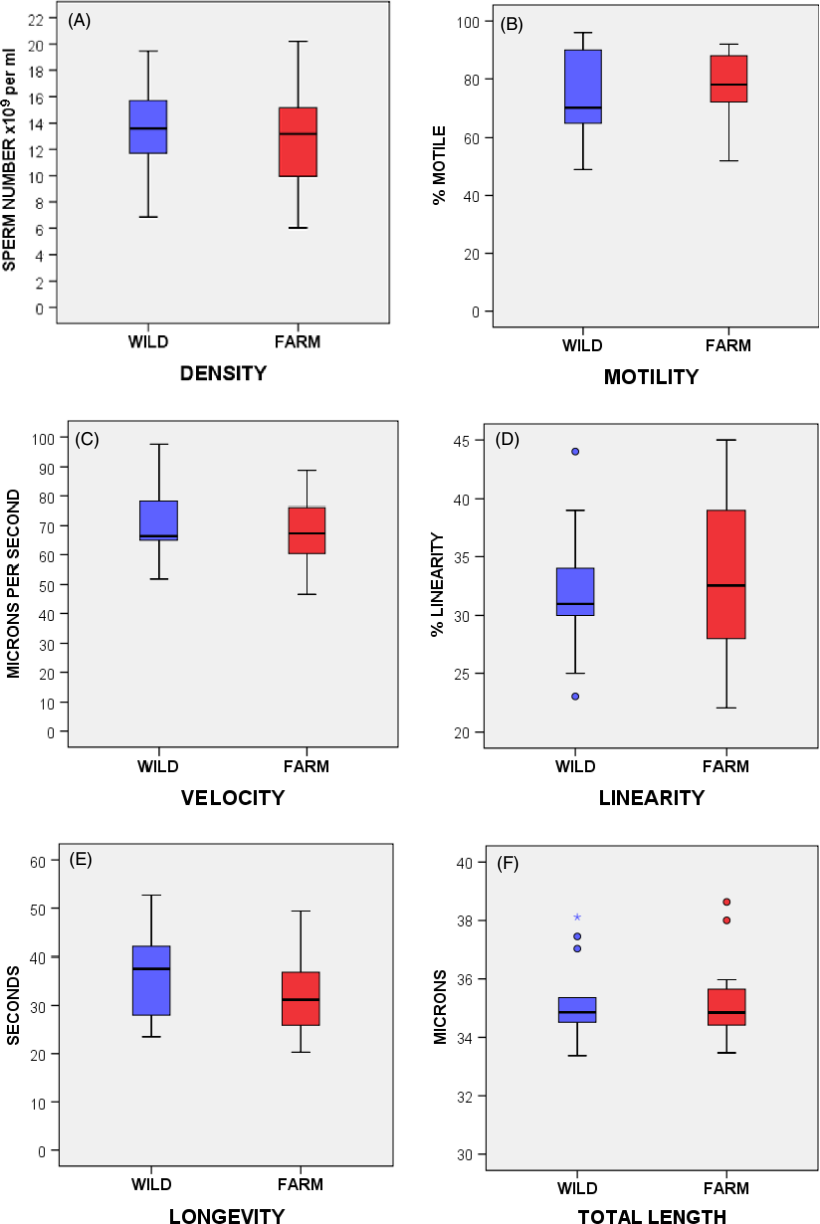
CASA measurements show equivalent sperm traits in wild and farm male Atlantic salmon. Boxplots showing medians (with quartile and interquartile ranges, outliers and extreme outliers) for six sperm trait measures: (A) density of sperm (×10^9^) per mL, (B) proportion of sperm showing progressive motility, (C) curvilinear swimming velocity, (D) linearity or path straightness (where 100 is perfectly straight), (E) duration of sperm motile lifespan and (F) sperm total length (*n *=* *18 + 18 males for each comparison except for sperm length (F) where *n *=* *14 + 14). See Results for for statistics.

We found no evidence that domestication had caused any changes to sperm form and function in comparisons between Aquagen farm and Namsen wild Atlantic salmon males (Fig. 1A–F). Farm fish showed similar sperm density per mL (*t*_1,34_ = 0.736, *P *=* *0.467) and sperm total length (*t*_1,26_ = 0.084, *P *=* *0.934) to wild fish, and sperm behaviour was no different with comparable proportions of sperm showing motility (*t*_1,34_ = −0.724, *P *=* *0.474), equal average sperm velocity (*t*_1,34_ = 0.655, *P *=* *0.517) and longevity (*t*_1,34_ = −0.757, *P *=* *0.454). Wild salmon sperm showed a more linear swimming path in river water, but the difference was not significant (*t*_1,34_ = 1.994, *P *=* *0.054), especially if applying Bonferroni correction to these multiple comparisons.

### Fertilization success

**Figure 2:**
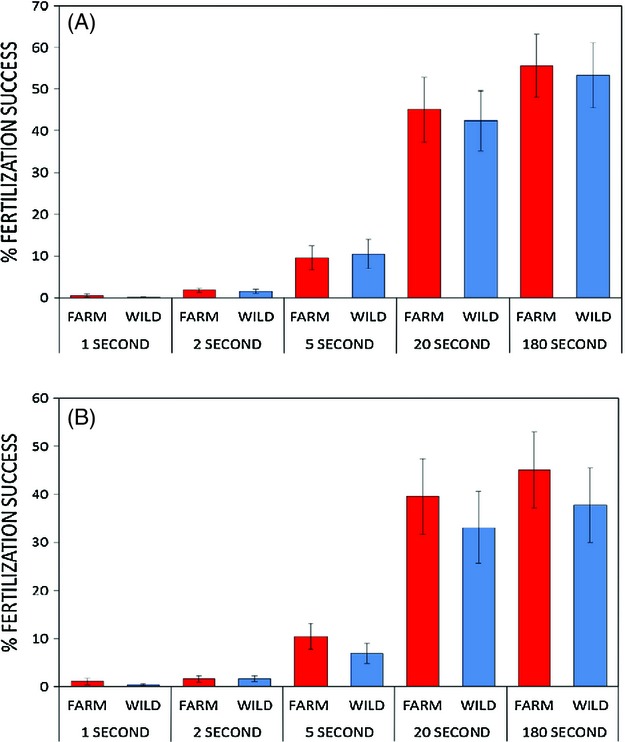
Mean fertilization rates (±1SE) of farm versus wild Atlantic salmon sperm (*n *=* *18 + 18 males) with either wild (A) or farm (B) eggs (from *n *=* *18 + 18 females), when given increasing gamete exposure times. Gamete exposure time showed significant differences between time treatments (see Results for for RM anova statistics).

Sperm from farm and wild males showed no difference in their ability to fertilize eggs. With an ample 180-s time period for sperm to find and fertilize ova (see mean fertilization success values on Fig. 2), sperm from farm and wild males showed similar levels of fertility, either with wild (*t*_1,17_ = 0.214, *P *=* *0.832) or farm eggs (*t*_1,17_ = 0.664, *P *=* *0.511).

When we applied a series of time limitation treatments to farm and wild salmon sperm, we still found no differences between the two strains in their ability to fertilize eggs from either farm or wild females (Fig. 2A, B). RM anova revealed a clear effect of increasing time of gamete exposure on fertilization success, but no differences between farm and wild males in their rates of fertilization. This was the case for trials with eggs from wild females (Fig. 2A), with a significant effect of gamete exposure time (*F*_4,17_ = 61.8, *P *<* *0.0001), but no difference between farm or wild male fertility (*F*_1,17_ = 0.183, *P = *0.674), and no strain x exposure time interaction (*F*_4,17_ = 0.169, *P = *0.954). We found similar results when testing with eggs from farm females (Fig. 2B; gamete exposure time: *F*_4,17_ = 37.08, *P *<* *0.0001; farm versus wild male fertility: *F*_1,17_ = 2.737, *P = *0.116; no strain x exposure time interaction: *F*_4,17_ = 1.271, *P = *0.29). Because fertilization data for the 1 s exposure time was not normally distributed, we reran the RM anova without this treatment, and results showed exactly the same pattern. Individual nonparametric Mann–Whitney analyses of *n *=* *18 farm versus *n *=* *18 wild male fertilization successes across each of the gamete exposure time blocks also showed that no differences existed between strains for either wild or farm eggs (maximum *Z *=* *0.728, minimum *P *=* *0.467).

### Female fertility and egg-sperm fertilization compatibility

**Figure 3:**
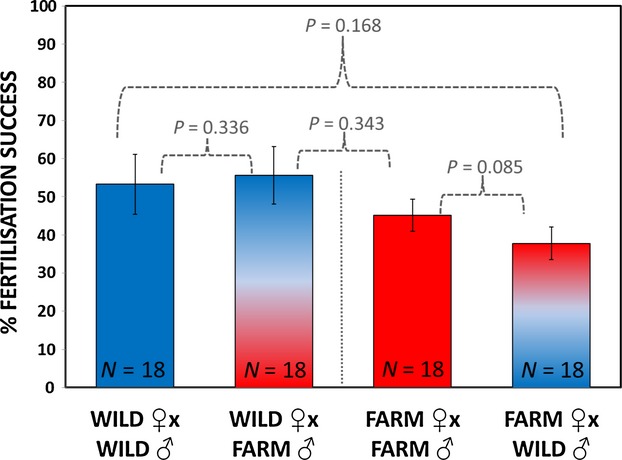
Fertility and fertilization compatibility comparisons (±1 SE) for either wild or farm eggs when fertilized by limited sperm doses from either farm or wild males. Results show no differences in relative egg fertility and no evidence for within-or between-strain egg-sperm compatibilities. Relative fertility comparisons were made using tests for independent samples, while compatibility comparisons were made within females using paired analyses (see Results for).

Farm female fertility was no different to that of wild salmon (Fig. 3), whether eggs were fertilized by sperm from wild males (*t*_1,34_ = 0.1.408, *P *=* *0.168) or with farm males (*t*_1,34_ = 0.962, *P *=* *0.343).

Overall, we also found no evidence that domestication had created fertilization incompatibilities within or between the farm and wild strains (Fig. 3). Using a paired *t-*test, where a female's egg fertility could be compared under similar conditions with sperm from either a male of her own strain versus sperm from the different strain, there was no difference in either condition (paired *t*_1,35_ = 0.781, *P *=* *0.44). If we analysed farm and wild females separately, there remained no indication that either strain's egg fertility depended on whether they were exposed to sperm from their own versus the different strain (wild females: paired *t*_1,17_ = 0.99, *P *=* *0.336; farm females: paired *t*_1,17_ = 1.83, *P *=* *0.085).

### Sperm competition success

**Figure 4:**
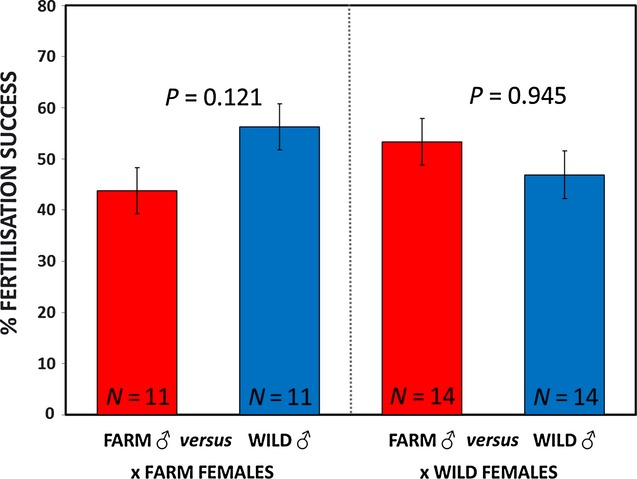
No differences in relative fertilization success for farm *versus* wild male sperm in competition for either farm or wild female eggs (means presented ± 1 SE). See Results for for analysis design and full statistics.

Farm males were no less competitive than wild males (Mann–Whitney *Z *= −1.032, *P *=* *0.302, *n *=* *25). Analysing female types separately revealed similar equivalence of farm and wild males in sperm competitions (Fig 4: competitions for farm females: *Z * =  −1.55, *P *=* *0.121, *n *=* *11; for wild females: *Z *= −0.069, *P *=* *0.945, *n *=* *14). Sperm density did not differ between farm and wild males (Fig. 1A), and across the 25 sperm competitions, average sperm density of farm/wild males was 0.51:0.49, with the maximum departure from this being 0.65:0.35 (farm/wild). The variance in relative sperm density between competing farm *versus* wild males did not covary with sperm competition success (competitions for wild females: *R *=* *0.164, *P *=* *0.569, *n *=* *14; competitions for farm females: *R *=* *0.375, *P *=* *0.255, *n *=* *11).

## Discussion

Following detailed experimental assessments, we find no differences between farm and wild Atlantic salmon in any measure of relative gamete performance. Farm Aquagen and wild Namsen Atlantic salmon show equivalence to each other in: (i) sperm form, function and number, (ii) egg and sperm fertility, (iii) sperm–egg compatibility and (iv) sperm competitiveness. As the adults used in our study were reared in a common hatchery environment, and as salmon from the Namsen were the main ancestral population to the Aquagen strain (Gjedrem et al. 1991; Garant et al. 2003), we can reject the hypotheses that relaxed selection, directed domestication and/or reduced genetic variation have led to any changes in farm Atlantic salmon sperm performance or relative egg fertility. Although we compared sperm function in river water, without additional ovarian fluid manipulations which can influence motility (e.g. Yeates et al. 2013, Beirão et al. 2014), our fertilization and sperm competition trials were run in the presence of either strain's ovarian fluid on their eggs, and therefore, these findings do account for any influence of ovarian fluid on reproductive performance. Moreover, comparisons of our hatchery-reared fish with Atlantic salmon that were born and raised in the wild reveal similarities in sperm densities and sperm length. Our hatchery fish had 12–13 × 10^9^ sperm cells per mL, equivalent to the 13.1 × 10^9^ mL ^−1^ found in precocious parr sourced direct from the wild (*n *=* *11), and a greater density than 6.1 × 10^9^ mL ^−1^ found in larger anadromous male salmon (*n *=* *66) that were captured from the wild then maintained for some weeks in hatchery tanks (Gage et al. 1995). Mean sperm length in Atlantic salmon raised in the wild varied between 32.3 and 39.5 μm (*n *=* *86) (Gage et al. 2002), equivalent to the 33.4–38.6 μm range we find in fish from the hatchery.

Although Atlantic salmon farming has been in operation for almost 50 years (Tilseth et al. 1991), the most likely explanation for our findings is that the period of domestication has not been sufficiently long, intense or misdirected, to enable altered or reduced genetic variation to act upon gamete function Instead, our findings show, at the gamete level, that escaped farm Atlantic salmon have clear and equal ability to reproduce with wild fish, even in the context of male–male competition. These findings will be relevant for assessing the risk and impact of farm salmon escapees in the natural environment. Evidence from an extensive genetic survey of 21 wild Norwegian Atlantic salmon populations indicates that, so far, farm salmon have had widely varying success at introgressing wild populations, despite adult farm escapees being recorded on the spawning grounds of every one of these river systems (Glover et al. 2012). In 6 of the 21 populations, evidence of introgression was recorded, with current salmon in the river Vosso and Opo showing 76% and 100% differences from prefarming genetic structures (Glover et al. 2012). Our findings for equivalent gamete performance in farm and wild males and females therefore support the belief that farm fish introgression is curtailed by whole-animal compromises to the behaviour and ecology of farm Atlantic salmon spawning and mating and/or lack of local adaptation. Detailed experimental work in enclosed natural streams and artificial spawning channels shows that farm Atlantic salmon show inferior reproductive performance, exacerbated in males by intrasexual competition (Fleming et al. 1996, 2000; Weir et al. 2004). As our measures were of gamete performance for farm fish derived from the same strain and rearing background as many of these studies, we can be confident that it was compromised spawning behaviour, and not inferior sperm or egg function, which explained the reductions in reproductive success.

The experimental studies conducted so far which recorded inferior levels of farm Atlantic salmon breeding performance have mainly compared farm fish direct from captivity with wild fish on their ascent to the spawning streams (Fleming et al. 1996, 2000; Weir et al. 2004). Some of the inappropriate or compromised behaviours by farm fish could therefore be the result of constrained development within a captive environment, which could impact upon reproductive behaviours. By contrast, in an experiment comparing the reproductive performance of farm and wild precocious male parr stages (an important reproductive strategy in Atlantic salmon, Jordan et al. 2007; Hutchings and Myers 1988), where all males were reared in a common hatchery environment, the farm males then showed much superior reproductive performance, compared with wild counterparts (Garant et al. 2003). Therefore, there remains an outstanding question as to whether the compromised reproductive performance of farm Atlantic salmon recorded in experimental studies is the result of environmental or genetic factors, and why such profound variability in introgression is measured between populations. If farm fish escape and then go through a more natural development to reproductive maturity before they ascend to the spawning beds (perhaps at a greater body size than wild relatives), then reproductive behaviour may become more appropriate, and our measures of gamete performance and compatibility indicate a clear ability to reproduce and then introgress.

Our findings of functional equivalence in the performance of farm salmon sperm and eggs under a range of tests add to concerns about the ability of farm Atlantic salmon to introgress wild populations, especially if the compromised breeding behaviour of farm fish straight from captivity can be improved by a period of readjustment in the wild before spawning. Improvements have already been made to the security of sea cages and farm containment to reduce the number of losses (Jensen et al. 2010), and this biosecurity should not be compromised. One solution to the problem of farm salmon introgression is through the production of fish that are reproductively sterile. The induction of triploidy through hydrostatic pressure on ova is routinely employed in some salmonids for wild stocking and farming (e.g. rainbow trout *Oncorhynchus mykiss*, Kozfkay et al. 2006; Piferrer et al. 2009). Triploid fish are usually infertile, or subfertile, reducing risks of introgression (Benfey 2009; Piferrer et al. 2009; Taylor et al. 2013), and the methods presented in our study here will provide meaningful assays for detailed testing of the fertility status of triploid fish. However, triploidy in farm Atlantic salmon has been resisted because the process can render stocks increasingly susceptible to cataracts and vertebral malformations (Piferrer et al. 2009; Taylor et al. 2013). Some studies have reported increased mortalities of triploid fish in freshwater or at sea (Galbreath and Thorgaard 1995; McGeachy et al. 1995; Cotter et al. 2002), and there are conflicting results that find reduced, equal or enhanced growth of triploids under aquaculture (e.g. Leclercq et al. 2011; Oppedal et al. 2003; Galbreath and Thorgaard 1995; Withler et al., 1995). Because of the prevalent risk and reported evidence of farm Atlantic salmon introgression, however, including our findings that farm salmon are reproductively equivalent at the gamete level, it seems likely that pressure will remain for Atlantic salmon farms to consider the use of triploid fish. A recent, carefully controlled study that compared diploid versus triploid mixed sibling groups across an entire commercial production cycle yielded important and relevant information on the application of triploidy for Atlantic salmon farming that will be safer for wild populations. Taylor et al. (2013) report overall growth rates to be equivalent between diploid and triploid fish, with triploids showing 30% faster growth in freshwater, but 7.5% slower growth at sea. Importantly, there was no difference in survival rates through the entire production cycle. Triploid individuals did show higher rates of cataract and skeletal deformations, both of which impact on fish welfare and commercial growth potential. Despite these negatives, however, the authors of this detailed study concluded that the potential for enhanced triploid growth, in conjunction with triploid specific diets and selective breeding for reducing the cataract and deformity problems, makes triploidy a genuine prospect for salmon aquaculture without the risks of introgression (Taylor et al. 2013). In the light of our findings for equivalent reproductive performance of farm *versus* wild Atlantic salmon, including within the relevant contexts of sperm competition and cryptic female choice (Birkhead et al. 2009), we suggest that triploid induction of sterility be more seriously considered as a route to prevent farm fish introgression, alongside more effective mechanisms to prevent escape or loss from farms (Jensen et al. 2010). Aquaculture is set to continue to grow, perhaps requiring genetic modification (Smith et al. 2010), so it will be important for biologists to identify, understand and quantify threats to wild systems and then work with the aquaculture industry for solutions that can balance rising global food demands against environmental protection.

## Statement on authorship

S.E.Y., S.E., I.A.F. and M.J.G.G. designed and ran the sperm trait assays, and fertilization and sperm competition trials. S.E.Y. conducted the paternity assays and W.V.H. helped with the computer-assisted sperm analyses. M.J.G.G. and S.E.Y. analysed the data and wrote the paper. All authors were involved in study design and contributed to the manuscript.

## Data archiving

Data sets for all experiments reported in this paper are available from the Dryad Digital Repository: http://dx.doi.org/10.5061/dryad.t72kr.
